# Efficacy of Polydeoxyribonucleotide in Promoting the Healing of Diabetic Wounds in a Murine Model of Streptozotocin-Induced Diabetes: A Pilot Experiment

**DOI:** 10.3390/ijms24031932

**Published:** 2023-01-18

**Authors:** Jiyoung Yun, SaeGwang Park, Ha Young Park, Kyung Ah Lee

**Affiliations:** 1 Department of Plastic and Reconstructive Surgery, Inje University Busan Paik Hospital, College of Medicine, Inje University, Busan 47392, Republic of Korea; 2 Department of Microbiology and Immunology, College of Medicine, Inje University, Busan 47392, Republic of Korea; 3 Innovative Therapeutic Research Institute, Inje University Busan Paik Hospital, College of Medicine, Inje University, Busan 47392, Republic of Korea; 4 Department of Pathology, Inje University Busan Paik Hospital, College of Medicine, Inje University, Busan 47392, Republic of Korea; 5 Department of Plastic and Reconstructive Surgery, Inje University Haeundae Paik Hospital, College of Medicine, Inje University, Busan 48108, Republic of Korea

**Keywords:** diabetes, wounds, healing, vascular endothelial growth factor, transforming growth factor-β1, collagen type I/III

## Abstract

We assessed the efficacy of polydeoxyribonucleotide (PDRN) in accelerating the healing of diabetic wounds in a murine model of streptozotocin (STZ)-induced diabetes. After the creation of diabetic wounds, the mice of the PDRN SC, PDRN IP and PBS groups received a subcutaneous, an intra-peritoneal injection of PDRN and a subcutaneous injection of PBS, respectively. After euthanasia, time-dependent changes in the wound diameter and histologic scores were measured and vascular endothelial growth factor (VEGF), transforming growth factor-β1 (TGF-β1) and collagen types I and III were assessed for their expression levels. The PDRN SC and the PDRN IP groups showed a significantly smaller diameter of diabetic wounds, significantly higher histologic scores, a significantly greater expression of VEGF, a significantly lower expression of TGF-β1 and a significantly greater expression of collagen types I and III as compared with the PBS group (*p* < 0.05 or 0.0001). In conclusion, PDRN might be effective in promoting the healing of diabetic wounds in a murine model of STZ-induced diabetes.

## 1. Introduction

Diabetes mellitus (DM) is defined as a group of metabolic derangements that are characterized by compromised insulin synthesis or function, which eventually results in hyperglycemia. DM is a serious condition, which has a great impact on the quality of life and survival of affected individuals. Consequences of hyperglycemia include risks of developing comorbidities that may affect several organs [1]. DM is one of the major chronic health problems worldwide. With prolonged life expectancy, its prevalence has increased [2]. Its global prevalence was estimated at 2.8% in the year 2000; it is expected to rise to 4.4% by 2030 [3]. The number of adult patients with DM is estimated at > 415 million worldwide, and its prevalence is increasing; more than 640 million adults are expected to be diagnosed with DM by 2040 [4]. DM is also one of the most prevalent diseases in Korea, which has been shown from the results of the nationwide health examination survey [5].

Patients with DM are vulnerable to chronic complications depending on the onset time and the degree of metabolic control. Common complications of DM include neuropathy (42.1%), retinopathy (44%), nephropathy (63.1%) and macroangiopathy (43%) [6]. Patients with DM are also vulnerable to peripheral neuropathy and peripheral vasculopathy of the lower extremities due to hyperglycemia, which are often neglected [7]. Occurrence of wounds in the lower extremities of these patients could make them susceptible to infections that may eventually lead to foot ulcers, thus termed diabetic foot ulcers (DFUs) [8]. DFUs are known as one of the most common complications of DM. Patients with DM are at 25% higher lifetime risk of developing diabetic foot as compared with their non-diabetic counterparts. Moreover, more than 50% of patients with diabetic foot develop intractable ulcers and then undergo amputation surgery [9]. Thus, patients with DM are at 17 and 15 times higher risk of developing DFUs and undergoing amputation surgery, respectively, as compared with their non-diabetic counterparts [10]. To date, early management and treatment protocols for diabetic foot have been well described in the literature [11,12]. However, there are no evidence-based guidelines for continuous surgeries after the initial surgery or amputations in patients with diabetic foot [13].

It is generally known that the process of wound healing (WH) typically includes four stages: hemostasis, inflammation, proliferation and remodeling. In patients with DM, however, this process is affected by hyperglycemia; they may exhibit a hypercoagulable state and decreased skin function during hemostasis; exacerbated inflammatory responses to chronic wounds because of derangements in inflammatory markers and growth factors (GFs); decreased proliferation of keratinocytes leading to insufficient wound re-epithelialization and an impairment in the involvement of fibroblasts in the synthesis, assembly and remodeling of the extracellular matrix (ECM) [8,14,15,16]. Thus, DM alters immune responses and lowers resistance to infection, thus causing diabetic wound infection, in which several mechanisms are involved [17,18]. Still, little is known about exact pathophysiologic mechanisms underlying an impairment in the WH in patients with DM. This poses a challenge for clinicians.

To date, studies have been conducted to induce or to treat DM in animal models. Rodents are often used for an animal model of DM because their advantages include small size, ease of maintenance, a sufficient length of life, good response to experimental factors, a capacity for good recovery and a high similarity to human anatomy and physiology [19,20]. It is generally known that animal models are used to examine the pathogenesis and pathophysiology of DM [21,22]. Over the past decades, both in vitro and in vivo studies have been conducted in the context of DM, although animal models still remain as useful tools for examining the complex characteristics of DM [23]. There are two major methods for creating an animal model of DM; these include disease induction and genetic manipulation, both of which are useful in clarifying specific mechanisms underlying its pathogenesis, pathophysiology and progression and extrapolating the experimental results to humans [24]. As one of the above two methods, streptozotocin (STZ) is a chemical inducer of type 1 or 2 DM in rodents; it targets pancreatic β-cells and thereby causes toxicity, including oxidation and hydroxylation [25,26,27,28].

Polydeoxyribonucleotide (PDRN) is low-molecular-weight DNA (50–1500 kDa) containing a mixture of deoxyribonucleotide polymers, with a chain length of 50–2000 bp. It is extracted from sperm cells of *Oncorhynchusmykiss* (salmon trout) or *Oncorhynchus keta* (chum salmon) at a purity of > 95% through purification and high-temperature sterilization [29,30,31]. To date, the effects of PDRN have been well described in the literature. In vitro studies have shown that PDRN promoted the growth of human fibroblasts and osteoblasts in primary cultures at therapeutic concentrations [30,31]. Moreover, in vivo studies have shown that PDRN stimulated WH in an experimental model of thermal injury, diabetes or ischemic skin flaps [32,33,34]. Furthermore, PDRN facilitated angiogenesis and neovascularization in an experimental model of peripheral artery occlusive disease [35]. Along the continuum of these previous literatures, the biological actions of PDRN in the context of DM have been documented [36,37]. Still, however, there is a paucity of data suggesting the efficacy of PDRN in the context of diabetic wounds in a murine model of STZ-induced diabetes.

Given the above background, we conducted this experimental study to assess the efficacy of PDRN in accelerating the healing of diabetic wounds in a murine model of STZ-induced diabetes.

## 2. Results

### 2.1. Gross Examinations of the Diabetic Wounds at 3, 7, 10 and 14 Days

**Figure 1:**
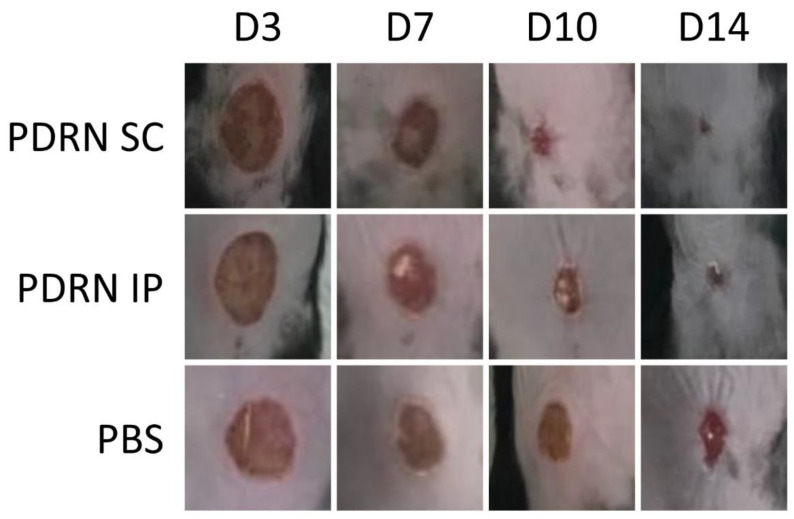
Gross examinations of the diabetic wounds were performed at 3, 7, 10 and 14 days. Then, time-dependent changes in the diameter of the diabetic wounds were monitored.

Gross examinations of the diabetic wounds were performed at 3, 7, 10 and 14 days. Then, time-dependent changes in the diameter of the diabetic wounds were monitored (Figure 1).

**Figure 2:**
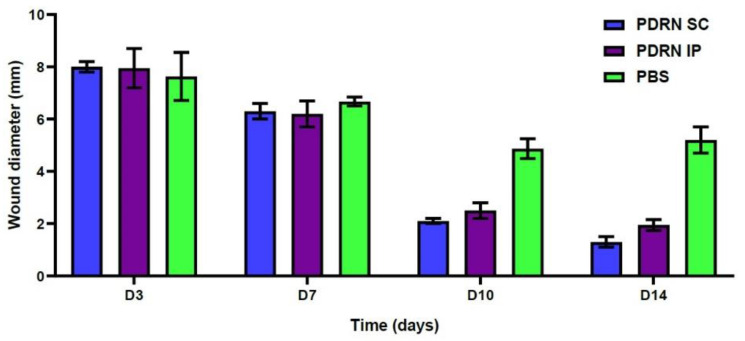
Time-dependent changes in the diameter of diabetic wounds at 3, 7, 10 and 14 days. At 10 days, the PDRN SC group showed a significantly smaller diameter of diabetic wounds as compared with the PBS group (2.1 ± 0.1 versus 4.87 ± 0.38 mm, respectively; *p* = 0.0024). Likewise, the PDRN IP group also showed a significantly smaller diameter of diabetic wounds as compared with the PBS group (2.5 ± 0.3 versus 4.87 ± 0.38 mm, respectively; *p* = 0.0053). At 14 days, the PDRN SC group showed a significantly smaller diameter of diabetic wounds as compared with the PBS group (1.3 ± 0.2 versus 5.2 ± 0.50 mm, respectively; *p* = 0.0002). Likewise, the PDRN IP group also showed a significantly smaller diameter of diabetic wounds as compared with the PBS group (1.95 ± 0.21 versus 5.2 ± 0.50 mm, respectively; *p* =0.0036).

At 10 days, the PDRN SC group showed a significantly smaller diameter of diabetic wounds as compared with the PBS group (2.1 ± 0.1 versus 4.87 ± 0.38 mm, respectively; *p* = 0.0024). Likewise, the PDRN IP group also showed a significantly smaller diameter of diabetic wounds as compared with the PBS group (2.5 ± 0.3 versus 4.87 ± 0.38 mm, respectively; *p* = 0.0053) (Figure 2).

At 14 days, the PDRN SC group showed a significantly smaller diameter of diabetic wounds as compared with the PBS group (1.3 ± 0.2 versus 5.2 ± 0.50 mm, respectively; *p* =0.0002). Likewise, the PDRN IP group also showed a significantly smaller diameter of diabetic wounds as compared with the PBS group (1.95 ± 0.21 versus 5.2 ± 0.50 mm, respectively; *p* = 0.0036) (Table 1) (Figure 2).

### 2.2. Histologic Examinations

**Figure 3:**
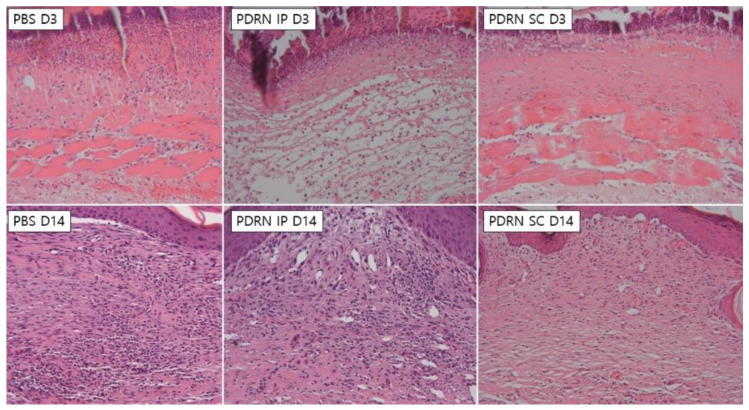
Histologic findings. In both the PDRN SC and the PDRN IP groups, there was a marked decrease in the number of inflammatory cells at 14 days as compared with 3 days. Moreover, at 14 days, the number of inflammatory cells was significantly smaller in both the PDRN SC and the PDRN IP groups as compared with the PBS group (hematoxylin and eosin, 200×).

In both the PDRN SC and the PDRN IP groups, there was a marked decrease in the number of inflammatory cells at 14 days as compared with 3 days (H&E, 200×) (Figure 3).

**Figure 4:**
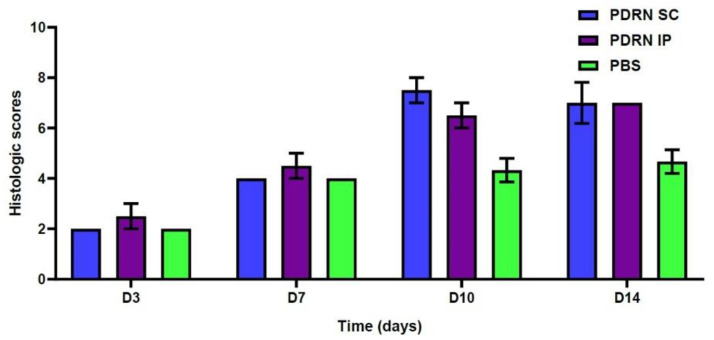
Histologic scores. At 10 days, the PDRN SC group had significantly higher histologic scores as compared with the PBS group (7.5 ± 0.5 versus 4.33 ± 0.47, respectively; *p* = 0.0055). Likewise, the PDRN IP group had significantly higher histologic scores as compared with the PBS (6.5 ± 0.5 versus 4.33 ± 0.47, respectively; *p* = 0.0158). At 14 days, the PDRN SC group had significantly higher histologic scores as compared with the PBS group (7.0 ± 0.82 versus 4.67 ± 0.47, respectively; *p* = 0.0248). Likewise, the PDRN IP group had significantly higher histologic scores as compared with the PBS group (7.0 ± 0.0 versus 4.67 ± 0.47, respectively; *p* = 0.0069).

At 10 days, the PDRN SC group had significantly higher histologic scores as compared with the PBS group (7.5 ± 0.5 versus 4.33 ± 0.47, respectively; *p* = 0.0055). Likewise, the PDRN IP group had significantly higher histologic scores as compared with the PBS group (6.5 ± 0.5 versus 4.33 ± 0.47, respectively; *p* = 0.0158). Moreover, at 14 days, the PDRN SC group had significantly higher histologic scores as compared with the PBS group (7.0 ± 0.82 versus 4.67 ± 0.47, respectively; *p* = 0.0248). Likewise, the PDRN IP group had significantly higher histologic scores as compared with the PBS group (7.0 ± 0.0 versus 4.67 ± 0.47, respectively; *p* = 0.0069) (Table 2, Figure 4).

### 2.3. Results of the Western Blotting Analysis

**Figure 5:**
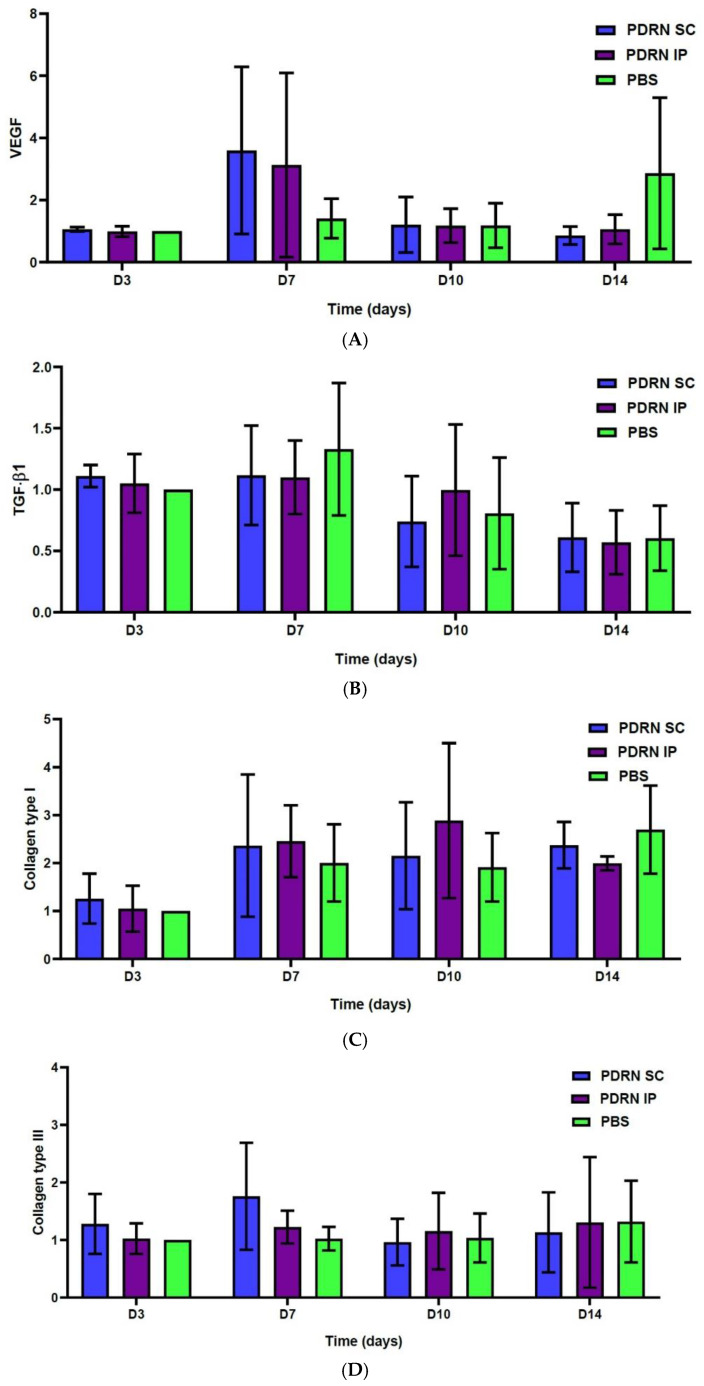
Results of the Western blotting analysis. (**A**) At 7 days, the PDRN SC group showed a significantly greater expression of VEGF as compared with the PBS group (*p* < 0.0001). Of note, at 7 days, the PDRN SC group showed a significantly greater expression of VEGF as compared with the PDRN IP group (*p* < 0.0001). (**B**) At 10 days, the PDRN SC group showed a significantly lower expression of TGF-β1 as compared with the PBS group (*p* < 0.0001). Of note, at 10 days, the PDRN SC group showed a significantly lower expression of TGF-β1 as compared with the PDRN IP group (*p* < 0.0001). (**C**) At 7 and 10 days, the PDRN SC and PDRN IP groups showed a significantly greater expression of collagen type I as compared with the PBS group (*p* <0.0001). (**D**) At 7 days, the PDRN SC group showed a significantly greater expression of collagen type III as compared with the PBS group (*p* < 0.0001).

At 7 days, the PDRN SC group showed a significantly greater expression of vascular endothelial growth factor (VEGF) as compared with the PBS group (*p* < 0.0001). Of note, at 7 days, the PDRN SC group showed a significantly greater expression of VEGF as compared with the PDRN IP group (*p* < 0.0001) (Figure 5A). At 10 days, the PDRN SC group showed a significantly lower expression of transforming growth factor-β1 (TGF-β1) as compared with the PBS group (*p* < 0.0001). Of note, at 10 days, the PDRN SC group showed a significantly lower expression of TGF-β1 as compared with the PDRN IP group (*p* < 0.0001) (Figure 5B). At 7 and 10 days, the PDRN SC and PDRN IP groups showed a significantly greater expression of collagen type I as compared with the PBS group (*p* < 0.0001) (Figure 5C). At 7 days, the PDRN SC group showed a significantly greater expression of collagen type III as compared with the PBS group (*p* < 0.0001) (Figure 5D).

## 3. Discussion

To date, PDRN has been employed to improve WH because it is equipped with an ability to promote the migration and growth of cells, the proper deposition of ECM and angiogenesis as well as to decrease inflammatory responses [38]. Advantages of PDRN include non-invasiveness, diverse routes of administration, strong therapeutic benefits, low immunogenicity and no toxicity. Use of PDRN has therefore been favored over conventional treatment modalities. This makes it possible to use PDRN even in compromised patients such as those with DM [32,38].

Molecular mechanisms underlying the efficacy of PDRN in promoting WH have been documented. Presumably, plasma membrane enzymes might be involved in the cleavage of PDRN. This serves as a source of purine and pyrimidine for diverse tissues. Moreover, nucleotides and nucleosides are generated from PDRN and thereby contribute to DNA synthesis, thus being involved in the activation of normal cell proliferation and growth [29]. This is closely associated with the effects of nucleotides and nucleosides on diverse types of GFs [39,40]. At therapeutic concentrations, PDRN raises the growth rate of diverse cells, such as fibroblasts, chondrocytes, preadipocytes and osteoblasts [41,42,43,44,45]. Briefly, PDRN has favorable effects on cell proliferation, possibly mediated by both the salvage metabolic pathways and the activation of adenosine receptors, which is characterized by the improvement of acute inflammation, upregulation of VEGF and promotion of granulation tissue formation [46].

Adenosine receptors belong to a group of essential neuromodulators; they play a key role in regulating diverse pathophysiological events [47]. These include adenosine A_1_, A_2A_, A_2B_ and A_3A_ receptors, which are expressed in immune or inflammatory cells [48,49]. Of these, the A_2A_ receptor is known to inhibit the release of pro-inflammatory cytokines [50,51]. PDRN serves as an A_2A_ receptor agonist that activates A_2A_ receptors; it exhibits anti-inflammatory effects by inhibiting the synthesis of pro-inflammatory cytokines (e.g., TNF-α and IL-6) [52,53]. Moreover, a decrease in the release of high-mobility group protein after treatment with PDRN has also been reported [53]. Furthermore, there was a marked increase in upstream VEGF signaling, accompanied by the increased expression of mature protein in wounds, after treatment with PDRN [37]. This justifies studies about the effects of PDRN on the healing of diabetic wounds.

The therapeutic effects of PDRN have been documented; it is effective in improving angiogenesis, promoting the activity of osteoblasts, stimulating the synthesis of collagen, accelerating the healing of burn wounds and inhibiting inflammatory responses [30,33,37,54,55,56,57]. Moreover, PDRN is effective in promoting the proliferation and migration of fibroblasts [58].

The efficacy of PDRN in improving diabetic wounds has been well described in the literature. According to an in vivo study using a murine model (db+/db+) of diabetes, PDRN was effective in healing an incisional skin wound based on the findings that there were significant increases in the wound breaking strength as well as the expression of VEGF and CD31 after a 12-day course of daily injection of PDRN [32]. Moreover, Kim et al. showed that PDRN was effective in improving both peripheral tissue oxygenation and angiogenesis in patients with diabetic ulcer [59].

To summarize, our results are as follows:

1. At 10 days, the PDRN SC group showed a significantly smaller diameter of diabetic wounds as compared with the PBS group (2.1 ± 0.1 versus 4.87 ± 0.38 mm, respectively; *p* = 0.0024). Likewise, the PDRN IP group also showed a significantly smaller diameter of diabetic wounds as compared with the PBS group (2.5 ± 0.3 versus 4.87 ± 0.38 mm, respectively; *p* = 0.0053).
2. At 14 days, the PDRN SC group showed a significantly smaller diameter of diabetic wounds as compared with the PBS group (1.3 ± 0.2 versus 5.2 ± 0.50 mm, respectively; *p* = 0.0002). Likewise, the PDRN IP group also showed a significantly smaller diameter of diabetic wounds as compared with the PBS group (1.95 ± 0.21 versus 5.2 ± 0.50 mm, respectively; *p* = 0.0036).
3. At 10 days, the PDRN SC group had significantly higher histologic scores as compared with the PBS group (7.5 ± 0.5 versus 4.33 ± 0.47, respectively; *p* = 0.0055). Likewise, the PDRN IP group had significantly higher histologic scores as compared with the PBS group (6.5 ± 0.5 versus 4.33 ± 0.47, respectively; *p* = 0.0158).
4. At 14 days, the PDRN SC group had significantly higher histologic scores as compared with the PBS group (7.0 ± 0.82 versus 4.67 ± 0.47, respectively; *p* = 0.0248). Likewise, the PDRN IP group had significantly higher histologic scores as compared with the PBS group (7.0 ± 0.0 versus 4.67 ± 0.47, respectively; *p* = 0.0069).
5. At 7 days, the PDRN SC group showed a significantly greater expression of VEGF as compared with the PBS group (*p* < 0.0001). Of note, at 7 days, the PDRN SC group showed a significantly greater expression of VEGF as compared with the PDRN IP group (*p* < 0.0001).
6. At 10 days, the PDRN SC group showed a significantly lower expression of TGF-β1 as compared with the PBS group (*p* < 0.0001). Of note, at 10 days, the PDRN SC group showed a significantly lower expression of TGF-β1 as compared with the PDRN IP group (*p* < 0.0001).
7. At 7 and 10 days, the PDRN SC and PDRN IP groups showed a significantly greater expression of collagen type I as compared with the PBS group (*p* < 0.0001).
8. At 7 days, the PDRN SC group showed a significantly greater expression of collagen type III as compared with the PBS group (*p* < 0.0001).

The above results suggest that subcutaneous or intra-peritoneal injections of PDRN might be effective in promoting the healing of diabetic wounds in a murine model of STZ-induced diabetes. At 10 days, however, the PDRN SC group showed a significantly lower expression of TGF-β1 as compared with the PDRN IP group and the PBS group (*p* < 0.0001). Presumably, this might be for several reasons. First, it has been previously shown that the degree of WH was maximized at 10 days after treatment with GFs [60]. Second, Reynolds et al. reported that WH was accelerated with re-epithelialization in association with upregulation of TGF-β1 [61]. Third, it has also been reported that the rate of absorption of a pharmacological agent is higher for an intra-peritoneal route as compared with a subcutaneous one [62]. 

However, our results cannot be generalized; limitations of the current study are as follows: First, we analyzed a small number of experimental mice. Second, we failed to assess the efficacy of an intra-lesional injection of PDRN in promoting WH. According to a review of the literature, an intra-lesional injection of PDRN has been assessed in the context of plastic and aesthetic surgery [63,64]. Further studies are therefore warranted to assess its efficacy in promoting the healing of diabetic wounds in a murine model of STZ-induced diabetes. Third, we failed to assess the efficacy of PDRN in inhibiting scar formation. Bitto et al. reported that PDRN was effective in lowering levels of pro-inflammatory mediators such as tumor necrosis factor-α (TNF-α), interleukin-6 (IL-6) and high-mobility group box protein-1 (HMGB-1) [65]. It can therefore be hypothesized that PDRN might be effective in inhibiting scar formation through down-regulation of inflammatory reactions and HMGB-1. According to Jeong et al., there were significant decreases in the scar area, inflammatory cell infiltration, the number of CD45-positive cells and the degree of expression of HMGB-1 after treatment with PDRN in a rat model of incisional WH. These authors finally suggested that the effects of PDRN in inhibiting inflammatory responses and promoting collagen synthesis through the suppression of HMGB-1 might contribute to accelerating WH and inhibiting scar formation [56]. Along the continuum of this previous experiment, two recent clinical studies have also advocated the efficacy of PDRN in the context of scar improvement [66,67]. Fourth, we failed to clarify a dose-response relationship. According to both in vitro and in vivo studies, approximately 100 mg/mL has been established as the optimal dose of PDRN in the context of cell growth, the expression of ECM protein and angiogenesis [38], but this deserves further study. Fifth, we failed to compare the efficacy of PDRN between combined and single administration routes in the current experiment. The diversity of administration routes is one of the advantages of PDRN therapy over conventional treatment modalities with limited injection routes. This is supported by not only experimental results that PDRN was effective in promoting the deposition of new sound tissue in wounds when administered at varying doses and via diverse administration routes from topical application to injection but also in vivo studies using similar administration routes, such as an intra-peritoneal injection or a topical application [38]. Further studies are therefore warranted to explore alternative administration routes in the context of collagen remodeling by fibroblasts.

Nevertheless, our results are of significance in that this is the first experiment to assess the efficacy of PDRN in promoting the healing of diabetic wounds in a murine model of STZ-induced diabetes even though its biological actions have been well described in the literature [56,68,69,70,71,72,73].

## 4. Materials and Methods

### 4.1. Experimental Animals and Setting

For the current experiment, we used a murine model of STZ-induced diabetes, as previously described [74,75]. For the current experiment, 5-week healthy male C57BL/6 mice weighing 22 g were purchased from a commercial vendor (Nara Biotech; Seoul, Korea) [76]. For ≥ 5 days prior to the experiment, 2–5 C57BL/6 mice were housed in a cage at a temperature of 24±1°C and a humidity of 55 ± 5%, with a 12-h (8:00 on 20:00 off) light–dark cycle under specific pathogen-free conditions. All mice were allowed free access to food and water. In the current experiment, all C57BL/6 mice were housed in these conditions. No other conditions were needed. the direct management of mouse feed was needed to measure the mice’s weight and the amount of their food intake. Moreover, diabetes-induced mice are characterized by frequent urination and excessive water intake. It was therefore mandatory to exchange a breeding cage and to check the amount of their water consumption in both the morning and afternoon [77,78]. This ensured that there would be no problems with the breeding of C57BL/6 mice. Thereafter, we established a murine model of STZ-induced diabetes; we used the C57BL/6 mice aged 8–12 weeks for further laboratory procedures.

The current animal study was conducted in compliance with the Animal Protection Law and the Laboratory Animal Act; its protocol was approved by the Institutional Animal Care and Use Committees (IACUC) of our medical institution (2021-001).

### 4.2. Establishment of a Murine Model of STZ-Induced Diabetes

STZ (55 mg/kg) was dissolved in a citrate buffer (pH = 4.5) and then intra-peritoneally injected in C57BL/6 mice aged 8–12 weeks once daily, five times consecutively. Establishment of a murine model of STZ-induced diabetes was confirmed when blood glucose levels exceeded 250 mg/dL at 7 days of the first injection. Once weekly thereafter, C57BL/6 mice’s tails were dissected and then evaluated for whether blood glucose levels were maintained at ≥ 250 mg/dL. At 4 weeks after the first injection, the C57BL/6 mice blood glucose levels were maximized and then remained constant. This was followed by the creation of wounds in a murine model of STZ-induced diabetes [79,80].

### 4.3. Rationale of the Estimation of the Number of Experimental Animals

For the current experiment, the number of C57BL/6 mice was estimated considering not only that ≥3 C57BL/6 mice per group were needed to perform a statistical analysis of the data but also that experimental procedures were performed in triplicate to confirm statistical significance. It is known that approximately ≤ 30% of C57BL/6 mice are dropped out of procedures because of death or experimental failure in an STZ-induced diabetes model. Therefore, the rate of success in inducing diabetes can be calculated as approximately 70%. Considering this, the initial number of C57BL/6 mice was divided by 0.7 [32]. 

We conducted the current experimental study to compare the degree of efficacy of PDRN between the administration routes in promoting the healing of diabetic wounds in a murine model of STZ-induced diabetes. We used HiDr Prefilled Inj. (BMI Korea, Cheongju, Chungcheongbuk-do, Korea) as PDRN; it is available as an injectable product only. Therefore, we planned to perform an experiment for three groups: PDRN SC, PDRN IP and PBS. In each group, after the treatment with PDRN via subcutaneous or intra-peritoneal routes or that with phosphate buffered saline (PBS) via a subcutaneous one, tissue samples were obtained at 3, 7, 10 and 14 days, followed by histologic examinations and Western blotting analysis. Three mice were needed at each time point. Therefore, a total of 12 C57BL/6 mice (n=12) were initially needed for each group.

However, considering a drop rate of 30% and that the experimental procedures were performed in triplicate, the total number of mice was finally estimated at [36/0.7] × 3 = 153 [32]. 

### 4.4. Dosing Rationale

According to a previous study, PDRN (8 mg/kg) showed no toxic effects on the brain, liver, lung, skeletal muscle or heart and it also caused no mortality [32]. Moreover, the commercially available PDRN has a concentration of 5.625 mg/3 mL [29]. Considering that the weight of a mouse is measured as 15–20 g, the total dose of PDRN couldbe calculated as 160 μL.

In the PDRN SC group, wounds were placed on the back; the experimental mice of the PDRN SC group received injections in the 3 and 9 o’clock directions at the margin of the diabetic wounds on the left and right sides of the back, respectively. Therefore, PDRN was injected in a total of four regions on the back. A 40 μL (3.75 mg/kg) amount of PDRN was injected in each region on the back. In the PDRN IP group, however, a total of 160 μL of PDRN was injected in a single dose, as previously described [81].

### 4.5. Rationale of Route of Administration

It is generally known that both chemical and physical properties of a substance and its dose and route of administration serve as determinants of its absorption, bioavailability and metabolism. Moreover, the route of administration is a critical factor that may determine the final pharmacokinetics, pharmacodynamics and the toxicity of pharmacological agents [82]. Specifically, the rate of absorption is the highest for the intravenous route, followed by the intra-peritoneal, intra-muscular, subcutaneous and oral routes, in decreasing order [62]. This explains why not only an intra-peritoneal route of administration is commonly used in small laboratory animals in which it is difficult to use an intravenous route but also why it is an alternative to an oral one for drugs that might be degraded in the gastrointestinal tract when orally administered [83].

A review of the literature has shown that PDRN has been administered via intra-peritoneal or subcutaneous routes in the context of WH [36,37,56,76,81]. Therefore, we have hypothesized that there might be a difference in the efficacy of PDRN in promoting diabetic wound healing between the two routes of administration.

### 4.6. Creation of Wounds

**Figure 6:**
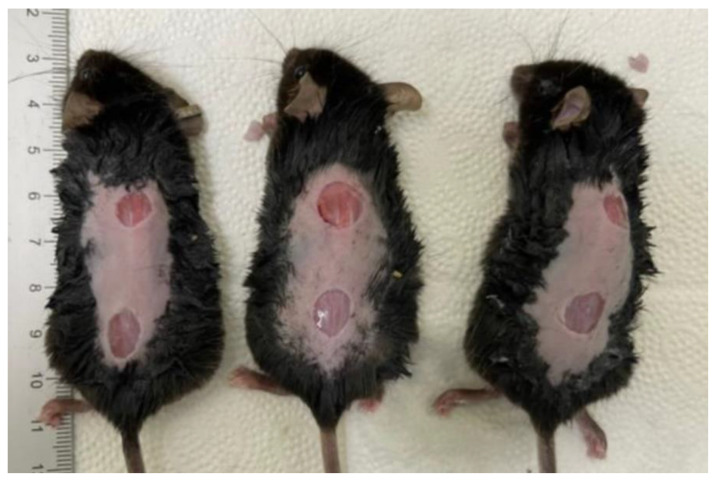
Creation of the diabetic wounds in C57BL/6 mice. Two circular wounds were created on the backs of mice using an 8-mm punch.

After removal of the hair on the dorsal skin of the mice using depilatory cream under general anesthesia, skin was disinfected with povidone-iodine solution and then washed with sterile water [84]. Two circular wounds were created on the backs of the mice using an 8mm punch and the skin was removed accordingly (Figure 6). Wound sites were disinfected with saline only because other types of disinfectants might affect their re-epithelialization [85,86]. Thus, oozing around wounds was removed and the adjacent region was also cleaned, but prophylactic antibiotics were not used because their use was not mandatory.

### 4.7. Experimental Procedures 

At day 0, wounds were created in a murine model of STZ-induced diabetes. At 1 and 2 days, the mice received injections of PDRN via either subcutaneous or intra-peritoneal routes. The mice of the PDRN SC group received a subcutaneous injection of PDRN in four regions on the back. In more detail, they received injections in the 3 and 9 o’clock directions at the margin of the diabetic wounds on the left and right sides of the back, respectively, once daily at a volume of 40 μL for each region. The mice of the PDRN IP group received an intra-peritoneal injection of PDRN once daily in a single dose, as previously described [81].

**Figure 7:**
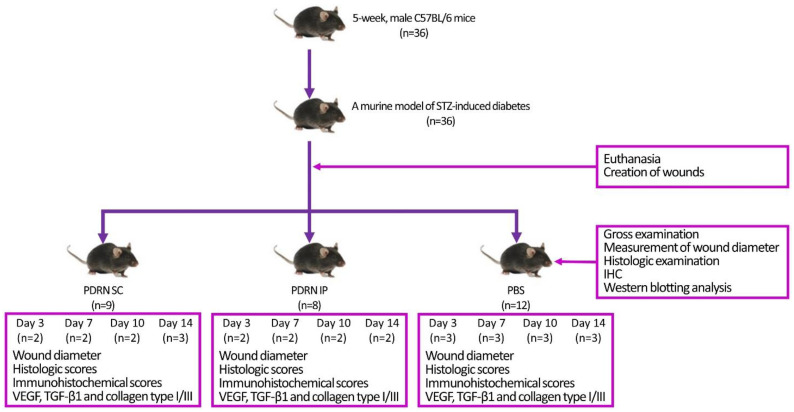
Experimental schema.

Ketamine and xylazine were mixed and then diluted at a ratio of 1/4 to set their concentration at 80 and 10 mg/kg, respectively. Then, a mixture of ketamine and xylazine was injected into the abdominal cavity and mice were euthanized accordingly [87]. Three mice were selected from each group, from which two tissue samples with a diameter of 12 mm, involving wounds, were obtained at 3, 7, 10 and 14 days. Of these, one tissue sample each was submitted for light microscopy and Western blotting analysis, respectively. For light microscopy, tissue samples were stained using H&E dye. Western blotting analysis was performed to identify specific proteins serving as markers of angiogenesis (e.g., VEGF and TGF-β1) and those serving as an indicator of the degree of wound recovery (e.g., collagen types I and III) [88,89]. An experimental schema is illustrated in Figure 7.

### 4.8. Evaluation and Criteria

Gross examinations of the diabetic wounds were performed at 3, 7, 10 and 14 days. Thus, time-dependent changes in the diameter of the diabetic wounds were monitored. 

We performed histologic examinations to analyze the degree of epithelialization, that of dermal matrix deposition and regeneration, that of granulation tissue formation and that of tissue remodeling. In each group, after the collection of tissue samples, a light microscopy was performed at a magnification of 200×. Then, histologic examinations were performed, whose total scores were calculated as previously described (Table 3) [32].

The current experiment also aimed to clarify differences in mechanisms underlying wound recovery. Therefore, we analyzed time-dependent changes in levels of VEGF, collagen type I/III and TGF-β1 in each group. Then, we compared the degree of angiogenesis and wound recovery between the three groups.

### 4.9. Statistical Analysis of the Experimental Data

All data was expressed as mean ± standard deviation (SD) or mean ± standard error of the mean (SEM), where appropriate. Differences in measurements between the three groups were analyzed using repeated measures of analysis of variance (ANOVA), followed by a *post-hoc* analysis. In each group, changes in measurements between the time points were analyzed using two-way ANOVA. All statistical analyses were performed using SPSS Windows version 15.0 (SPSS Inc., Chicago, IL, USA) or GraphPad Prism 6.0 software (GraphPad Software, Inc., San Diego, CA, USA). A *p*-value of < 0.05 was considered statistically significant.

## 5. Conclusions

In conclusion, our results indicate that PDRN might be effective in promoting the healing of diabetic wounds in a murine model of STZ-induced diabetes, but this deserves further clinical study.

## Figures and Tables

**Table 1:** Diameter of diabetic wounds.

Variables	Values											
	PDRN SC (n = 9)				PDRN IP (n = 8)				PBS (n = 12)			
	3 Days (n = 2)	7 Days (n = 2)	10 Days (n = 2)	14 Days (n = 3)	3 Days (n = 2)	7 Days (n = 2)	10 Days (n = 2)	14 Days (n = 2)	3 Days (n = 3)	7 Days (n = 3)	10 Days (n = 3)	14 Days (n = 3)
Diameter (mm)	8.0 ± 0.2	6.3 ± 0.30	2.1 ± 0.1	1.3 ± 0.2	7.95 ± 0.75	6.2 ± 0.50	2.5 ± 0.3	1.95 ± 0.21	7.63 ± 0.92	6.67 ± 0.17	4.87 ± 0.38	5.2 ± 0.50

Abbreviations: PDRN, polydeoxyribonucelotide; SC, subcutaneous; IP, intra-peritoneal. Values are mean ± standard deviation.

**Table 2:** Histologic scores.

Variables	Values											
	PDRN SC (n = 9)				PDRN IP (n = 8)				PBS (n = 12)			
	3 Days (n = 2)	7 Days (n = 2)	10 Days (n = 2)	14 Days (n = 3)	3 Days (n = 2)	7 Days (n = 2)	10 Days (n = 2)	14 Days (n = 2)	3 Days (n = 3)	7 Days (n = 3)	10 Days (n = 3)	14 Days (n = 3)
Histologic scores	2.0 ± 0.0	4.0 ± 0.0	7.5 ± 0.5	7.0 ± 0.82	2.5 ± 0.5	4.5 ± 0.5	6.5 ± 0.5	7.0 ± 0.0	2.0 ± 0.0	4.0 ± 0.0	4.33 ± 0.47	4.67 ± 0.47

Abbreviations: PDRN, polydeoxyribonucelotide; SC, subcutaneous; IP, intra-peritoneal. Values are mean ± standard deviation.

**Table 3:** Criteria for calculating histologic scores.

	Criteria	
	Epidermal and Dermal Regeneration	Granulation Tissue Thickness
1	Little epidermal and dermal organization	Thin granulation layer
2	Moderate epidermal and dermal organization	Moderate granulation layer
3	Complete remodeling of epidermis and dermis	Thick granulation layer
4		Very thick granulation layer

## Data Availability

The data presented in this study are available on request from the corresponding author.
